# Antibody-conjugated species-sorted single-walled carbon nanotubes for multiplexed cytokine sensing

**DOI:** 10.1038/s43246-026-01169-y

**Published:** 2026-04-27

**Authors:** Amelia K. Ryan, Sadiyah Parveen, Zachary Cohen, Atara Israel, Ryan M. Williams

**Affiliations:** 1 The City College of New York, Department of Biomedical Engineering, New York, NY USA; 2 Stony Brook University, Department of Medicine, Division of Nephrology & Hypertension, Stony Brook, NY USA

**Keywords:** Biosensors, Biosensors

## Abstract

Single-walled carbon nanotubes (SWCNT) are versatile building blocks for optical sensors. Their near infrared photoluminescent emission is determined by their chiral structure. Commercially available SWCNT are polydisperse, leading to spectral congestion and only single analyte sensing. Aqueous two-phase extraction (ATPE) is a technique to sort SWCNT structures for improved nanosensor performance and multiplexed sensing. To date, ATPE has not been demonstrated with bioconjugate-compatible surface chemistries. Here, we demonstrate improved and multiplexed cytokine sensing by ATPE sorting SWCNT species with amine-functionalized ssDNA. We show that this approach enables effective chirality sorting and direct antibody conjugation. We used sorted, functionalized SWCNT to simultaneously and specifically detect the inflammatory cytokines IL-12 and IL-6 with high sensitivity and selectivity, and improved robustness compared to unsorted SWCNT. We anticipate that such sensitive multiplexed sensors will be useful for implantable and bedside diagnostics of inflammatory processes while serving as a model for sensing other complex biological processes.

## Abstract (web summary)

Aqueous two-phase extraction is used for sorting single-walled carbon nanotubes with primary amine functionalized DNA, enabling molecularly-specific multiplexed sensing.

## Introduction

Single-walled carbon nanotubes (SWCNT) are highly effective transducers for optical sensors. They emit near infrared (NIR) narrow-bandgap photoluminescence dependent on the chiral angle of the sp^2^ carbon lattice helix, a feature conducive to biomedical sensing applications^1^. Each species is denoted by an (*n,m*) index defined by this chiral angle and tube diameter.

Though SWCNT possess such useful inherent properties, some amount of processing is necessary for these applications. As-produced SWCNT are hydrophobic and thus readily aggregate^2^. Non-covalent functionalization with DNA is a widely used for solubilizing SWCNT as it disperses individual nanotubes in aqueous media due through π-π base stacking on the SWCNT surface and charge repulsion of the phosphate backbone^3–5^. Such non-covalent solubilization allows for the bright, stable photoluminescence of SWCNT in solution, as well as imparting biocompatibility^6,7^. Long-term in vivo studies have shown that SWCNT wrapped with DNA show no signs of short-term or chronic toxicity^6^.

Synthesis of SWCNT-DNA constructs has allowed for further screening and development, with or without the addition of biomolecular recognition elements, as effective biosensors. Many studies have utilized SWCNT-DNA to detect a wide variety of analytes: small molecules such as H_2_O_2_, and doxorubicin, neurotransmitters including dopamine, norepinephrine, and serotonin, and protein biomarkers ranging from gynecologic cancer-associated proteins to inflammatory cytokines^8–13^. Several studies have used functionalized DNA oligonucleotides to anchor molecularly specific probes to the SWCNT surface without compromising the optical properties of the SWCNT. Other studies have used DNA as a base to conjugate further sensor elements. In particular, primary amine-functionalized DNA (SWCNT-DNA-NH_2_) is necessary to attach an antibody to the nanotube construct^14,15^. Carbodiimide crosslinking has been used to form an amide bond between the DNA-NH_2_ and the antibody, creating a stable bond and molecular-specific nanobiosensor. This antibody-based approach has been used to target ovarian cancer biomarker HE4, prostate cancer biomarker uPA, the estrogen receptor, and inflammatory cytokine interleukin-6 (IL-6) with high specificity^14–16^.

Commercially available SWCNT are comprised of many unique (*n,m*) species, and thus have many, often-overlapping, optical transitions^17–19^. Circumventing such spectral overlap in SWCNT transducer NIR photoluminescence is necessary to produce discrete, highly sensitive sensors, as well as to produce highly-multiplexed sensors^20^. SWCNT chiral sorting and purification is an enabling step towards these goals^21^. Several effective separation techniques developed over the past two decades include density gradient centrifugation, gel chromatography, ion exchange chromatography, and aqueous two-phase extraction (ATPE)^18,22–24^.

ATPE is widely-used for nanomaterials separation in general, and SWCNT separation specifically, as it is versatile, scalable, and relatively low-cost^18,25–34^. Some ATPE separations used bile salt surfactants to solubilize SWCNT prior to sorting and subsequent surfactant exchange with DNA^26,35–37^. Others used DNA to solubilize SWCNT prior to sorting that was subsequently used directly in sensing applications^38–41^. This is analogous to prior ion-exchange chromatography separation methods, which relied on ssDNA recognition of individual (*n,m*) species^24^. However, this sorting method has thus far limited chirally-pure SWCNT sensor development to analytes that can be detected with the same sequence used for chirality sorting. Further functionalization of SWCNT-DNA sorted through ATPE, such as the above-mentioned carbodiimide cross-linking with antibodies has yet to be realized.

In this work, we demonstrated ATPE sorting of SWCNT with primary amine-functionalized DNA, which enabled subsequent molecularly-specific multiplexed sensing for the first time. We evaluated separation patterns, side-by-side, of both non-functionalized and aminated versions of several known ATPE-active DNA sequences. We found that the ATPE method is indeed robust, with some pattern differentiation, to functional and non-functional DNA sequence incorporation. We then performed antibody conjugation directly to the purified SWCNT-DNA-NH_2_, producing the first multiplexed SWCNT sensors functionalized with commercial biomolecular recognition elements—in this case for the inflammatory cytokines IL-6 and IL-12. These targets were chosen due to their crucial roles in cell-mediated immunity. IL-6 and IL-12 regulate both acute and chronic inflammation and are valuable biomarkers of inflammatory diseases^42,43^. The framework presented here could be applied to a wide variety of targets as the vast library of commercially available antibodies only broadens the utility of this straightforward multiplexed sensor construction method.

## Results and discussion

### ATPE sorting of SWCNT-DNA-NH_2_

**Fig. 1:**
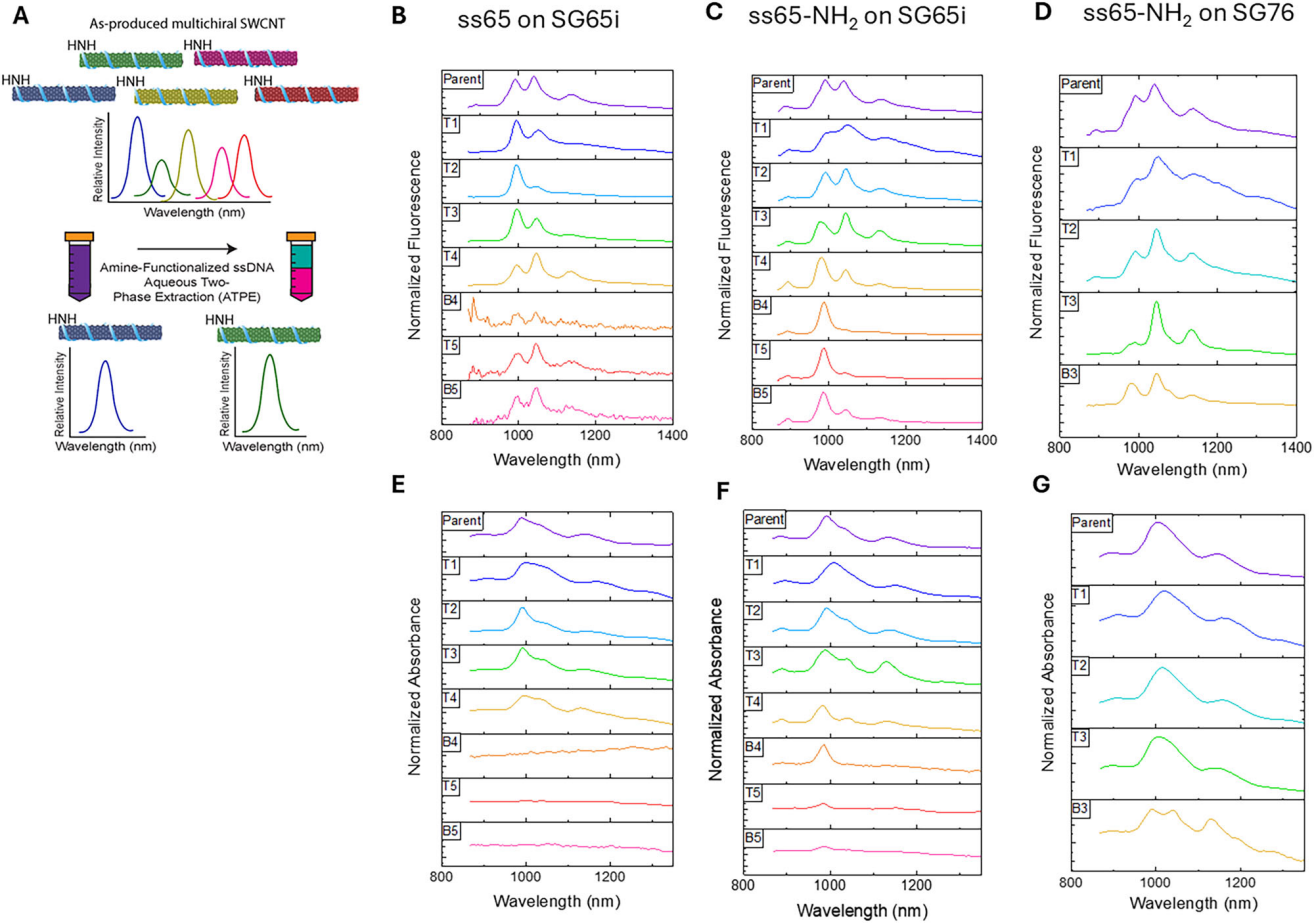
ATPE sorting with ss65-NH_2_. **A** Diagram of workflow. **B** Fluorescence spectra acquired with 638 nm laser from each phase of ATPE sorting SG65i SWCNT using ss65. **C** Fluorescence spectra from each phase of sorting SG65i SWCNT using ss65-NH_2_. **D** Fluorescence spectra from each phase of sorting SG76 SWCNT using ss65-NH_2_. **E** Absorbance spectra from each phase of ATPE sorting SG65i SWCNT using ss65. **F** Absorbance spectra from each phase of sorting SG65i SWCNT using ss65-NH_2._ **G** Absorbance spectra from each phase of sorting SG76 SWCNT using ss65-NH_2_.

The (6,5) Super Sequence, or ss65, is a known recognition sequence for (6,5) SWCNT^31^. As expected, it enabled purification of the (6,5) chirality from SG65i-prepared SWCNT stock (Fig. 1A, B, E), wherein the T2 Fraction demonstrates strong (6,5) purification. However, separation of SWCNT with NH_2_-functionalized ss65 exhibited markedly different behavior while still being able to achieve separation. (6,5) nanotubes encapsulated with ss65-NH_2_ appear more resistant to partitioning to the top phase in that they require more polyvinylpyrrolidone (PVP) to move to the top phase in comparison to their non-aminated counterparts (Figs. 1C, 1F). Although it required a higher number of extractions (and more PVP) to achieve the same purity, ss65-NH_2_ also enabled (6,5) purification in the B4 and T5 fractions. The final yield of the SWCNT sorted with ss65 and ss65-NH_2_ were roughly the same.

One potential explanation for this observation is differences in hydrophilicity of the SWCNT-ss65 and the SWCNT-ss65-NH_2_. The amine group contains a nitrogen atom with a lone pair of electrons that can form hydrogen bonds with water molecules—increasing the hydrophilic character of the DNA strand^44^. We hypothesize that this may increase the overall hydrophilicity of the resulting SWCNT constructs, in turn making each (*n,m*) species more likely to partition to the hydrophilic bottom phase of the ATPE system^31^. Therefore, more PVP partitioning modulator is needed to extract the SWCNT-DNA-NH_2_ from the bottom phase compared to the non-aminated SWCNT-DNA^18,28,31^. This difference in hydrophilicity may enable modified and potentially even enhanced ATPE sorting resolution. It also suggests that functional groups on DNA that affect the SWCNT construct’s solvation energy, such as a primary amine group, could be used to fine-tune ATPE resolution. However, we note that while amine functionalization alters ATPE partitioning behavior, it does not improve chiral purity, as the main rationale for sorting with functionalized ssDNA is for compatibility with downstream chemical functionalization. This is the first instance of chemically-modified DNA enabling ATPE sorting of SWCNT. It demonstrates the robustness of the ATPE method, but also the possibility for further customization of chirality-sorted SWCNT through the use of DNA with other functional groups. It will be necessary to perform future work to investigate whether chemically modified DNA impacts the purity and/or resolvability of the ATPE SWCNT sorting.

We further assessed the ability of amine-functionalized DNA to enable SWCNT chirality sorting using SG76-prepared SWCNT, also using the ss65-NH_2_ sequence. In this study, iterative additions of PVP to the ATPE system enabled a (7,5)-sorted fraction to emerge in T3 (Fig. 1D, G). These results suggest that the robustness of the ATPE method is preserved when using amine-functionalized DNA to sort multiple chiralities. Although these experiments yielded chirality-sorted fractions of SWCNT-DNA-NH_2_, the concentration of SWCNT in the final products was relatively low. Thus, we sought to assess DNA-NH_2_-enabled sorting scale-up as previously demonstrated with ATPE^31,34,45^.

In order to achieve sufficient quantities of purified SWCNT-DNA-NH_2_, we scaled up the separation by a factor of eight. We observed similar purities of (6,5) achieved in the scaled sorting system (Supplementary Figs. 1A, 2A). We also investigated the use of ss76 as it has been reported as a (7,5) recognition sequence in ion exchange chromatography^28^. However, when used in the ATPE sorting system, ss76-NH_2_ enabled gradual extraction of almost all chiralities except (7,6) from SG76 nanotubes, leaving purified (7,6) in the bottom phase (Supplementary Figs. 1B, 2B). While we found minor variations between the results of the 400 μL and 3200 μL systems, the sorting behavior of the scaled-up system was largely predictable. For our applications, we prioritized high-yield samples, using (6,5)-enriched fractions that contain no (7,6) and (7,6)-enriched fractions that contain no (6,5), rather than completely monochiral samples with lower yield. The scaled-up system yielded sufficient amounts of purified SWCNT for our antibody conjugation process, though we anticipate that larger scales could be used for future applications.

Additionally, we attempted the surfactant exchange method to attach DNA-NH_2_ to SWCNT, which had been previously sorted using bile salt surfactants as an alternate route to obtain the base constructs for antibody-conjugated sensors^27,29,32^. This method resulted in severe aggregation and loss of SWCNT fluorescence and, therefore, was not utilized. We speculate that the surfactant exchange method may not be suitable for aminated DNA due to the influence of the primary amine group on the DNA’s polar character.

### Antibody conjugation

**Fig. 2:**
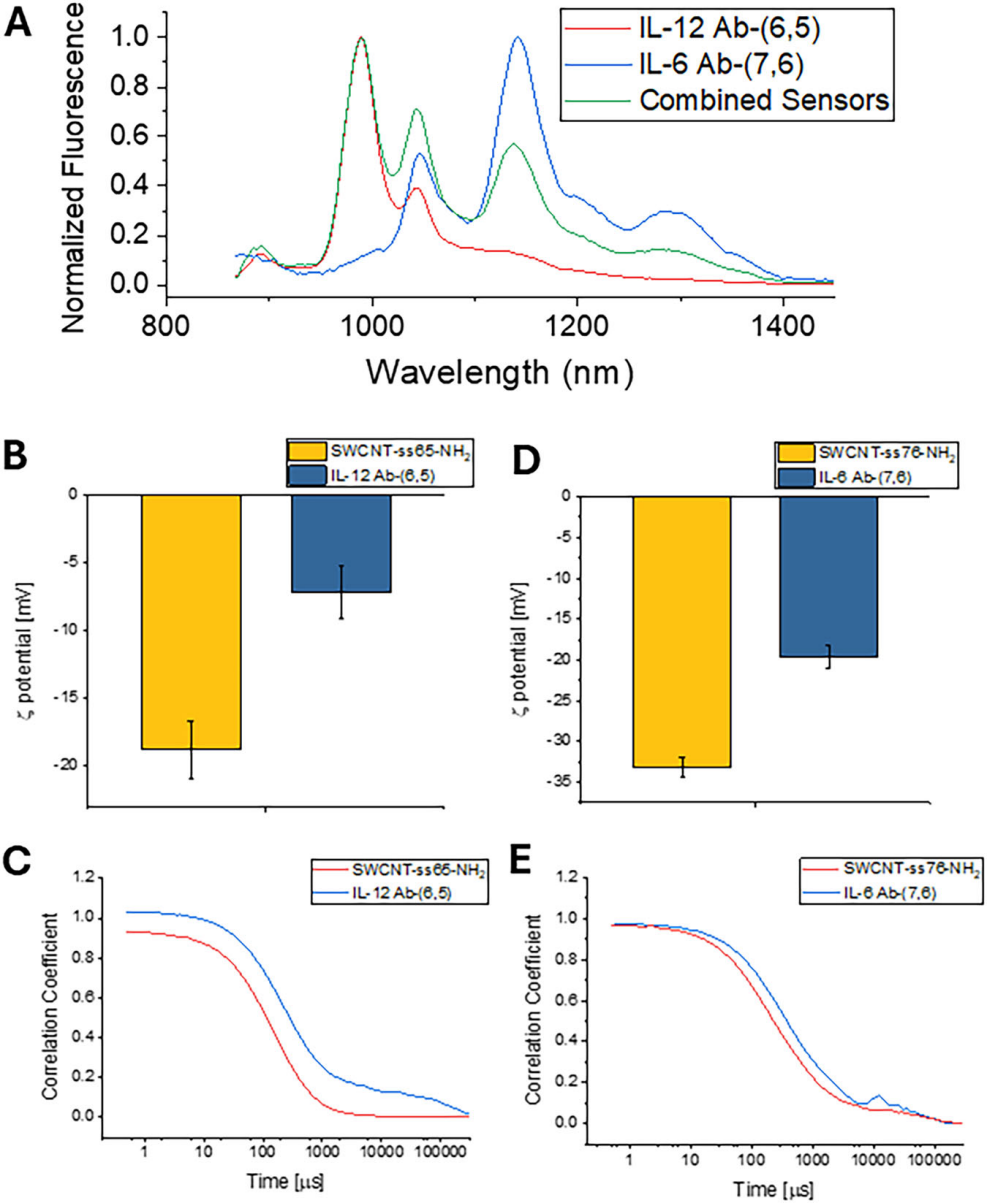
Optical characterization of antibody-conjugated nanosensors. **A** Representative fluorescence spectra acquired with 638 nm laser of antibody-conjugated nanosensors before deployment. **B** Electrophoretic light scattering indicates (6,5) SWCNT construct increased in charge after IL-12 antibody conjugation. **C** Electrophoretic light scattering indicates (7,6) SWCNT construct increased in charge after IL-6 antibody conjugation. **D** Dynamic light scattering indicates (6,5) SWCNT construct increased in size after IL-12 antibody conjugation. **E** Dynamic light scattering indicates (7,6) SWCNT construct increased in size after IL-6 antibody conjugation.

To demonstrate the potential of multiplexed SWCNT-based sensors, we incorporated a standard antibody conjugation methodology with the chirality-enriched SWCNT. We conjugated an anti-IL-12 antibody to (6,5)-enriched SWCNT to produce IL-12 Ab-(6,5) and anti-IL-6 to the (7,6)-enriched SWCNT to produce IL-6 Ab-(7,6). Optical characterization revealed that the SWCNT constructs retained their fluorescence signal after antibody conjugation (Fig. 2A). When the sensors were combined, both the (6,5) and (7,6) chiralities show pronounced peaks, demonstrating that these SWCNT can be used for spectral multiplexing without overlap. Although there is a considerable (7,5) peak in the fluorescence measurement of our combined sensors, we found that this did not inhibit the function of our multiplexed sensor system. Further sensor characterization confirmed successful antibody conjugation as the relative size and ζ-potential of the SWCNT constructs increased, which indicates successful conjugation (Fig. 2B–E)^14–16,46^.

**Fig. 3:**
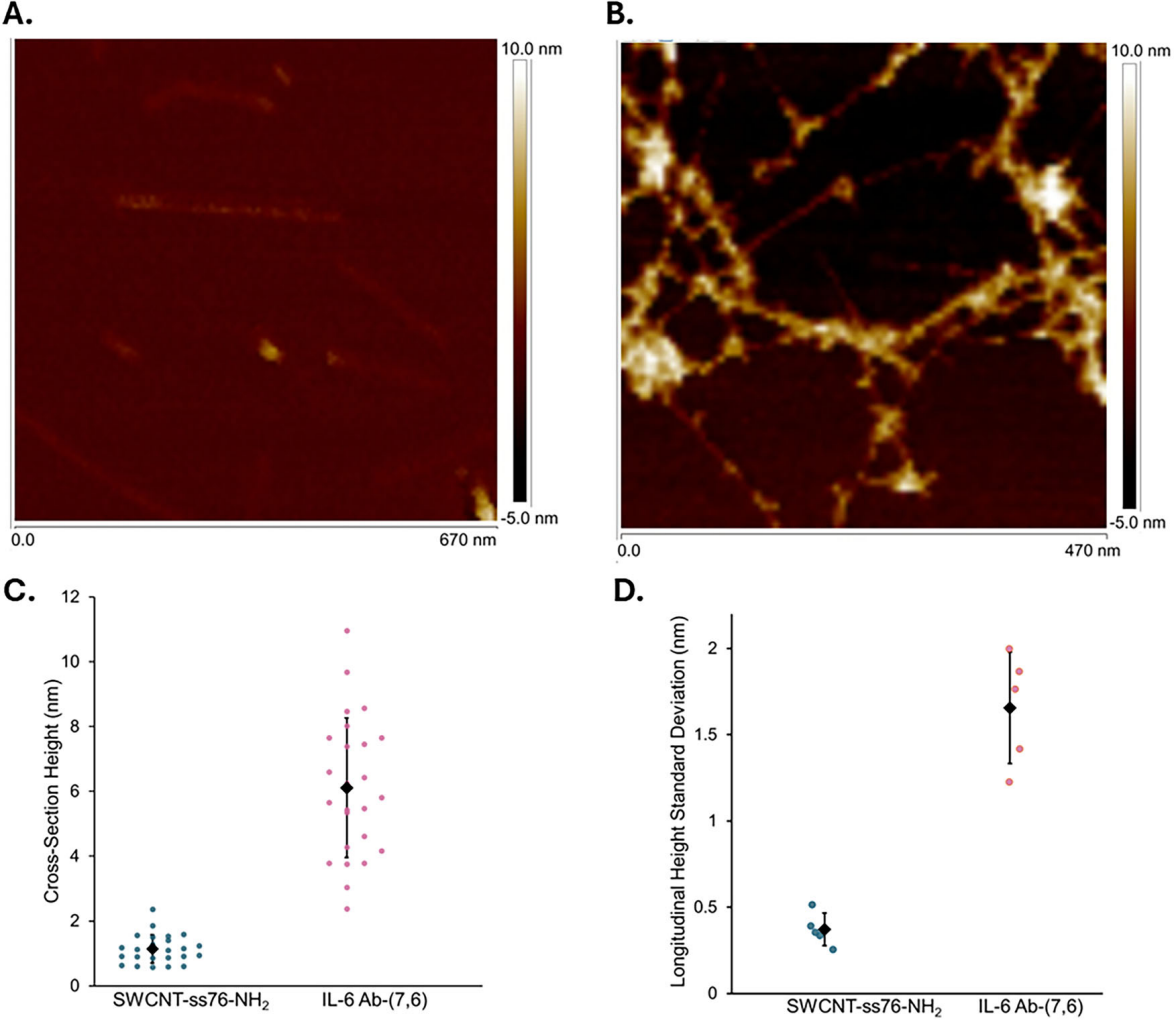
Structural validation of antibody conjugation by AFM. **A** Representative AFM height image of SWCNT-ss76-NH_2_ deposited on mica. **B** Representative AFM height image of antibody-conjugated IL-6 Ab-(7,6) SWCNT. **C** Quantification of perpendicular cross-sectional heights (*n* = 25, Welch’s two-tailed t-test, *p* = 1.53E-11). **D** Longitudinal height variability quantified as the population standard deviation of height along visually isolated nanotube segments (*n* = 5, Welch’s two-tailed t-test, *p* = 0.000487).

To further confirm successful antibody attachment at the single-nanotube level, atomic force microscopy (AFM) was performed on SWCNT-ss76-NH_2_ and antibody-conjugated IL-6 Ab-(7,6) constructs (Fig. 3A, B, Supplementary Fig. 3). Perpendicular cross-sectional analysis revealed a significant and biologically relevant increase in nanotube height following antibody conjugation. SWCNT-ss76-NH_2_ exhibited cross-sectional heights of 1.14 ± 0.438 nm, consistent with individually dispersed ssDNA-functionalized nanotubes. Reported AFM heights and known diameters for individual SWCNT typically fall between **~**0.5 and 1.5 nm, while DNA-wrapped nanotubes generally exhibit apparent heights of ~1–2 nm depending on sequence and substrate interactions^47–49^. In contrast, IL-6 Ab-(7,6) displayed significantly increased heights of 6.11 ± 2.15 nm (Fig. 3C). In both cases, measurements avoided visible SWCNT aggregates induced by filtration-based concentration and sample drying on the imaging surface. The magnitude of this increase is consistent with AFM studies of biomolecule-functionalized carbon nanotubes that report several-nanometer height profile additions that correspond to attached proteins^50,51^. It also corresponds very well to known IgG antibody dimensions, which are ~15 nm in height, 10 nm in width at the Fv region, and 4 nm in width at the Fc region^52–54^.

Longitudinal height profiling further revealed increased surface heterogeneity following conjugation. SWCNT-ss76-NH_2_ exhibited relatively uniform height along the nanotube backbone (longitudinal standard deviation: 0.37 ± 0.09 nm), whereas IL-6 Ab-(7,6) displayed pronounced protrusions and significantly increased longitudinal height variability (1.66 ± 0.33 nm) (Fig. 3D, Supplementary Fig. 3). These results indicate heterogeneous, non-uniform antibody decoration along the nanotube surface rather than formation of a continuous coating.

Longitudinal height profiles exhibited discrete, spatially separated increases in height profile consistent with localized antibody attachment. Prior studies have reported an approximate one-to-one mass ratio of ssDNA to SWCNT, indicating that multiple ssDNA strands coat a single nanotube and provide numerous potential conjugation sites^15,55^. Consistent with this, the observed raised profile suggests that multiple antibodies are distributed along individual SWCNT. However, steric hindrance and reaction kinetics likely limit achievable surface coverage. We therefore find that approximately 2-3 antibodies are present per 100 nm SWCNT, consistent with a prior studies with scFV fragment conjugation to SWCNT^50,51^. Together, these observations support successful antibody conjugation to SWCNT.

While previous ATPE studies have seen higher purity with strictly monochiral samples, in this work, we chose to focus on achieving functional purity with higher yield, which is necessary for downstream conjugation, purification, and testing. With two high-yield samples with non-overlapping peaks (i.e. a (6,5)-rich sample that contains no measurable (7,6) and a (7,6)-rich sample that contains no measurable (6,5)), we were able to efficiently construct two antibody-conjugated sensors with bright, non-interfering emission peaks. Future work may seek to optimize the sorting process of SWCNT-DNA-NH_2_ to achieve even higher chemical purity without sacrificing yield.

There have been several examples of carbodiimide crosslinking performed to attach antibodies to aminated DNA on SWCNT for optical sensing, all of which have used unsorted SWCNT and have exclusively used (TAT)_6_-NH_2_ as the chosen DNA sequence^14–16^. Our successful conjugation of antibodies to ATPE-sorted fractions in this work establishes that chirality-sorted SWCNT can also serve as base constructs for antibody-conjugated sensors. Furthermore, we have shown that the antibody conjugation reaction can be performed in environments containing polyethylene glycol (PEG) and dextran (DEX) solutions. This work also demonstrates that aminated DNA sequences other than (TAT)_6_-NH_2_ can be used for this application.

Other works have utilized SWCNT-DNA for ATPE sorting and subsequent sensing with no further biomolecular recognition element functionalization^38–41^. However, these sensing applications have been limited to analytes that can be detected using non-selective DNA sequences used for ATPE sorting. Other studies have circumvented this limitation by sorting surfactant-dispersed SWCNT, then switching out the surfactant for the DNA sequence of choice after ATPE is completed^26,35–37^. Our work here provides a more direct and versatile alternative: sorted SWCNT fractions from ATPE can be immediately functionalized with any antibody of choice. The vast library of antibodies allows for virtually any biomarker to be targeted. The direct route to functionalization improves efficiency and prevents loss of carbon material. These new developments open a myriad of possibilities in the world of SWCNT research.

### Multiplexed sensing with chirality-sorted SWCNT

**Fig. 4:**
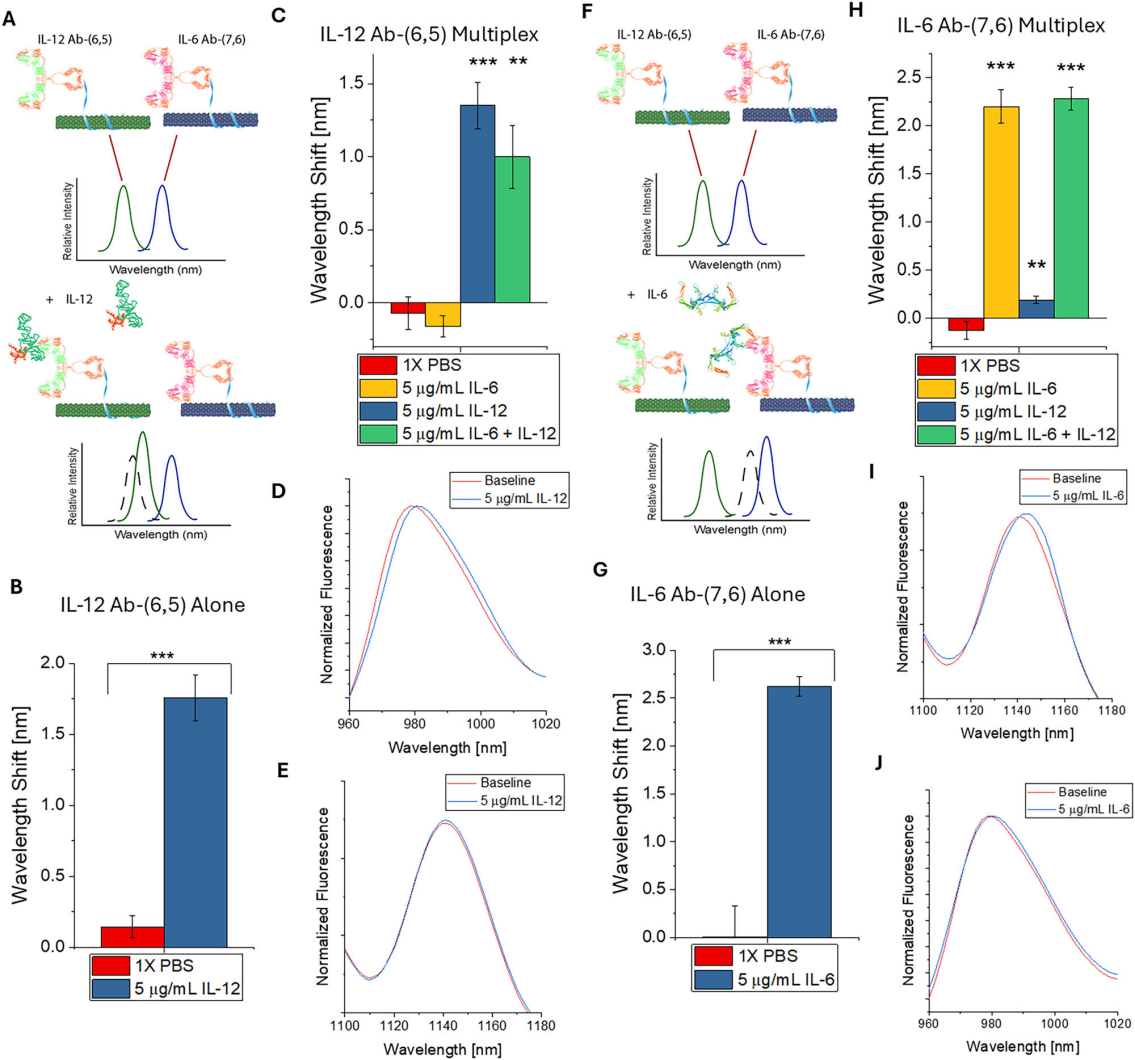
Multiplexed cytokine detection using chirality-sorted SWCNT-antibody sensors. **A** Schematic of anticipated (6,5) peak response to IL-12. **B** Wavelength shift of IL-12 Ab-(6,5) alone in response to 5 µg/mL IL-12 protein (*p* = 0.000103). **C** Wavelength shift of (6,5) peak in combined sensor samples response to cytokine addition (acquired with 655 nm laser) (IL-6: *p* = 0.475. IL-12: *p* = 0.000286. IL-6 + IL-12: p = 0.00274). **D** Representative (6,5) spectral shift from combined sensors in response to IL-12 only. **E**) Representative (7,6) spectral shift from combined sensors in response to IL-12 only. **F** Schematic of anticipated (7,6) peak response to IL-6. **G** Wavelength shift of IL-6 Ab-(7,6) alone in response to 5 μg/mL IL-6 protein (*p* = 0.000184). **H**) Wavelength shift of (7,6) peak in combined sensor samples response to cytokine addition (IL-6: *p* = 0.0000361. IL-12: *p* = 0.0210. IL-6 + IL-12: *p* = 0.0000967). **I** Representative (7,6) spectral shift from combined sensors in response to IL-6 only. **J**) Representative (7,6) spectral shift from combined sensors in response to IL-12 only. Mean ± standard deviation; two-tailed t-test for all.

After chirality purification and antibody conjugation, we anticipated that IL-12 Ab-(6,5) would enable a shift in the (6,5) peak in response to IL-12 protein (Fig. 4A). When tested individually, IL-12 Ab-(6,5) exhibited an average red shift of 1.76 nm +/− 0.16 nm after 3 hours of exposure to 5 μg/mL IL-12 protein (Fig. 4B). After confirming the basic functionality of IL-12 Ab-(6,5), we combined it with IL-6 Ab-(7,6) to assess simultaneous function. IL-12 Ab-(6,5) exhibited a red shift in response to samples containing IL-12 only and IL-12 in addition to IL-6 (Fig. 4C, Supplementary Figs. 4, 5). Importantly, the (6,5) peak showed a significant red shift in response to IL-12 protein, while the (7,6) peak did not (Fig. 4D, E). These results demonstrate the ability of the chirality-sorted analyte-specific sensors to function independently of each other.

Similarly, we anticipated that IL-6 Ab-(7,6) would enable a shift in the (7,6) peak in response to IL-6 protein (Fig. 4F). When tested individually, IL-6 Ab-(7,6) exhibited an average red shift of 2.62 nm +/− 0.10 nm in response to 5 μg/mL IL-6 protein (Fig. 4G). When both sensors were combined, IL-6 Ab-(7,6) showed a robust red shift in response to samples containing IL-6 alone as well as IL-6 and IL-12 together (Fig. 4H, Supplementary Fig. 4, 6). As expected, the (7,6) peak shifts in response to IL-6 protein alone while the (6,5) peak does not (Fig. 4I, J). In samples where both cytokines are present, both (6,5) and (7,6) peaks show a red shift (Supplementary Fig. 4, 7). We also analyzed the visible (7,5) peak, which was apparent in both the (6,5)- and (7,6)-sorted samples, finding no significant response to cytokine challenges (Supplementary Fig. 8). This may have been due to interferent binding events if (7,5) recognizing both IL-12 and IL-6 were present. Or it may simply be that the (7,5) is poorly responsive to this type of analyte, as we have previously demonstrated some chiralities are less responsive than others in antibody-conjugated SWCNT sensors^14–16^.

To evaluate the function of the antibody and potential role of nonspecific binding in the observed response, unconjugated SWCNT-DNA-NH_2_ were also tested against IL-6 and IL-12. As expected from our prior studies^14–16^, the passivated SWCNT constructs without antibodies did not exhibit a significant shift in response to either cytokine (Supplementary Fig. 9).

To further validate the potential of this system, we sought to independently detect two separate cytokines, IL-8 and IL-1β. We performed an identical separation and conjugation process as above, wherein we sought to detect only IL-8 with the (6,5) chirality and only IL-1β with the (7,6) chirality. These sensors exhibited an almost identical performance, with each sorted peak shifting in response to its designated cytokine (Supplementary Fig. 10). This further validation is a clear demonstration of the versatility of this separation-conjugation and the chirality-sorted SWCNT-ssDNA-NH_2_ as a base construct for detecting many possible biomarkers simultaneously.

Here, we demonstrate spectral multiplexing using two different SWCNT chiralities in the same solution for the first time. The use of ATPE-purified SWCNT allows us to clearly interpret the optical signal of each chirality with minimal interference from other chiralities, metallic SWCNT, defective SWCNT, and other impurities. We have shown here that SWCNT of different chiralities can be sorted, functionalized separately against unique targets, and then combined into one sample to create a sensor array in which each chirality represents sensors against a different target.

**Fig. 5:**
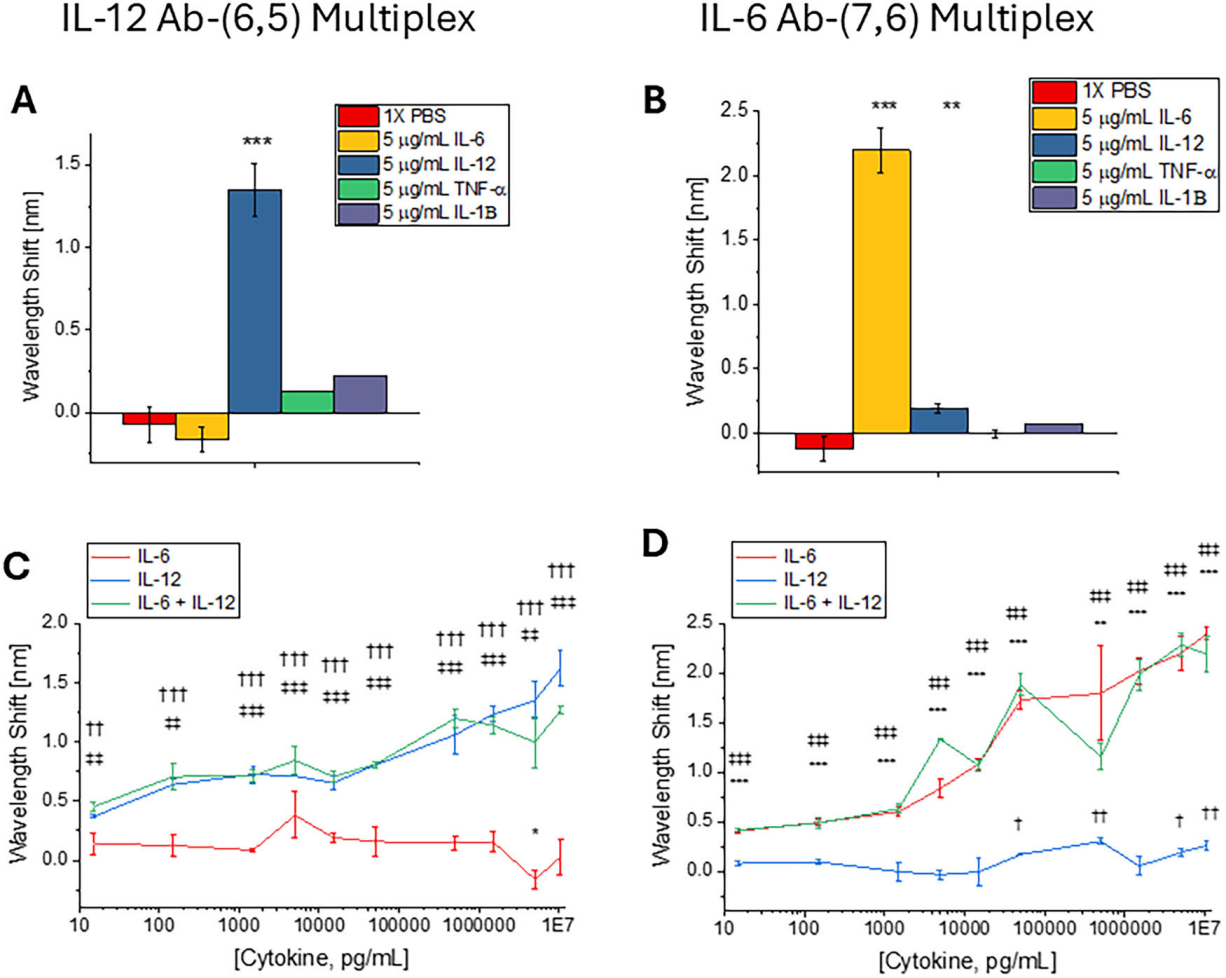
Selectivity and sensitivity of the multiplexed nanosensor. **A** Wavelength shift of (6,5) peak in response to IL-6 (*p* = 0.475), IL-12 (*p* = 0.000286), TNF-α (*n* = 2, *p* = 0.761), and IL-1β (*n* = 1, *p* = 0.154). **B** Wavelength shift of (7,6) peak in response to IL-6 (*p* = 0.0000361), IL-12 (*p* = 0.0210), TNF-α (*n* = 2, *p* = 0.129), and IL-1β (*n* = 1, *p* = 0.574). **C** Concentration-response curve of the (6,5) peak (acquired with 655 nm laser) (*p* values in Table S2). **D** Concentration-response curve of the (7,6) peak (p values in Table S3). Mean ± standard deviation; two-tailed t-test vs. control with no analyte for all.

We performed an additional challenge to the specificity of the sensors by testing them against inflammatory cytokines interleukin-1β (IL-1β) and tumor necrosis factor-α (TNF-α), two immunologically similar signaling molecules. Neither the (6,5) nor the (7,6) peak showed any significant shifts in response to IL-1β or TNF-α, further supporting the high molecular specificity of these sensor constructs (Fig. 5A, B).

We sought to further understand the functionality of the sensor array by testing its dynamic range and limit of detection (LOD). The sensors were again deployed against IL-12, IL-6, and both cytokines combined at concentrations from 10 μg/mL to 15 pg/mL, a wide range of physiologically relevant cytokine levels. Even when titrated to as low as 15 pg/mL, both (6,5) and (7,6) peaks showed significant wavelength shifts in response to their respective cytokine. The (6,5) peak exhibited 0.368 nm red shift (+/− 0.013 nm) in response to 15 pg/mL IL-12. The (7,6) peak exhibited 0.407 nm red shift (+/− 0.029 nm) in response to 15 pg/mL IL-6. Both peaks exhibited similar patterns of response to their target cytokine and the combined cytokine samples. 15 pg/mL IL-12 + IL-6 yielded 0.452 nm (+/−0.039 nm) from the (6,5) peak and 0.416 nm (+/ 0.0095 nm) from the (7,6) peak (Supplementary Tables 2, 3). Neither peak showed considerable shifts in response to their non-target cytokine alone (Fig. 5C, D). These results indicate that the response of the (6,5) peak is caused by the IL-12 antibody binding event, the response of the (7,6) peak is caused by the IL-6 antibody binding event, and that the sensor response is not additive.

The concentrations at which we demonstrated detection are well within the physiologically relevant ranges for both cytokines—healthy levels of IL-6 in human serum are reported from 0 to 181 pg/mL, while patients with inflammatory disease have elevated IL-6 serum levels. 567 pg/mL has been observed in Alzheimer’s disease patients, 692 pg/mL in rheumatoid arthritis patients, while extremely high levels—up to 500,000 pg/mL—are reported in cases of severe sepsis^56–59^. Fewer studies have been published on clinically relevant levels of IL-12. Some report healthy patients do not show serum IL-12 levels higher than 5 pg/mL (the typical sensitivity limit of ELISA), while others cite healthy serum levels at 82 pg/mL. However, upper limits of IL-12 levels in serum have been reported as 164.3 pg/mL in major depressive disorder patients, 442.7 pg/mL in chronic hepatitis B patients, and 371.2 pg/mL in malaria patients^57,60–63^. We note that, in our study, detection as such small concentrations is enabled by the small variation in sensor center wavelength shifts, likely afforded by the uniformity of the separated SWCNT transducers used.

In our literature search, we found no previously published optical sensors and only two examples of electrochemical sensors for IL-12 with LODs of 500 pg/mL and 3.5 pg/mL^64,65^. Literature on IL-6 sensors is much more extensive. Previously published sensors for IL-6 show a wide range of detection limits depending on their modality. Electrochemical sensors can detect IL-6 in the low pg/mL range, with one electrochemical impedance sensor detecting IL-6 in serum at a low limit of 0.01 fg/mL^58,66^. Optical sensors use surface plasmon resonance, fluorescence, and surface-enhanced Raman scattering to achieve LODs as low as 1.1 pg/mL, 0.02 pg/mL, and 0.028 pg/mL, respectively^67–70^. Previously published SWCNT optical sensors for IL-6 have reported LODs of 3.91 μg/mL and 105 ng/mL using corona phase molecular recognition and aptamer-based approaches, respectively^12,71^. Our group has reported detection as low as 25 pg/mL using the same antibody-based design as the present study, but with polydisperse SWCNT rather than chirality-enriched^16^.

**Fig. 6:**
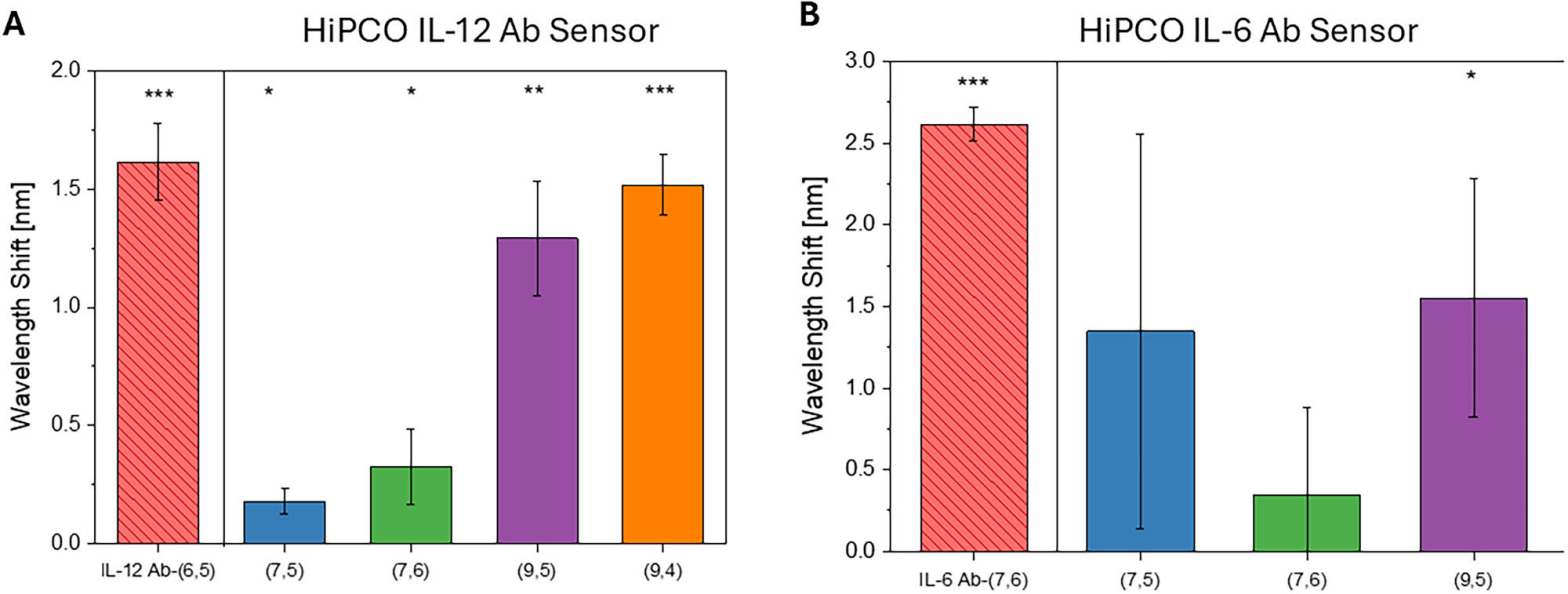
Chirally-pure sensor comparison to bulk chirality sensors. **A** Wavelength shifts of IL-12 Ab-(6,5) (left) and anti-IL-12-conjugated HiPCO SWCNT (right) in response to 5 μg/mL IL-12 protein. (acquired with 638 nm laser) (IL-12 Ab-(6,5): *p* = 0.000103. (7,5): *p* = 0.0149. (7,6): *p* = 0.0366. (9,5): *p* = 0.00158. (9,4): *p* = 0.000153). **B** Wavelength shifts of IL-6 Ab-(7,6) (left) and anti-IL-6-conjugated HiPCO SWCNT (right) in response to 5 μg/mL IL-6 protein (IL-6 Ab-(7,6): *p* = 0.000184. (7,5): 0.131. (7,6): *p* = 0.374. (9,5): *p* = 0.0321). Mean ± standard deviation; two-tailed t-test vs. control with no analyte for all.

We then sought to assess the contributions of chirality-purified SWCNT to sensor robustness and repeatability. We compared chirally-pure SWCNT sensors to bulk chirality SWCNT sensors using the same antibody recognition element. Multi-chiral HiPCO SWCNT functionalized with the anti-IL-12 exhibited a significant red shift in response to recombinant IL-12 protein for all chiralities analyzed, though the degree of shifting varies among chiralities (Fig. 6A). The (6,5) peak is not visible in the HiPCO mixture at the laser excitation wavelength used, so this peak cannot be directly compared to IL-12 Ab-(6,5). The mean wavelength shift exhibited by the (9,5) chirality in the HiPCO sensors (1.79 nm) is similar to the mean shift exhibited by IL-12 Ab-(6,5) (1.76 nm) in the same testing conditions. However, the standard deviation of the chirality-sorted sensors is lower (0.244 nm in HiPCO sensors, 0.164 nm in (6,5) sorted sensors). There is also less shifting and smaller variation in the control group of IL-12 Ab-(6,5) compared to the unsorted HiPCO sensors, indicating less signal interference in the chirality-sorted SWCNT. Multi-chiral HiPCO SWNCT functionalized with anti-IL-6 were also tested against 5 μg/mL IL-6 protein in 1X PBS. The (7,5) and (7,6) chiralities did not exhibit significant wavelength shifts, while the (9,5) chirality showed a mean shift of 1.55 nm with a standard deviation of 0.73 nm (Fig. 6B). The shift observed in (9,5) is both lower in magnitude and less statistically significant than the shift observed in IL-6 Ab-(7,6). The large standard deviation observed in the comparison to bulk sensors can be attributed to noisy signal due to spectral congestion and overlap, while the chirality-sorted sensors see less deviation due to fewer overlapping peaks.

These results support the long-held hypothesis that chirality sorting of SWCNT improves optical sensor performance. The chirality-sorted sensors demonstrated less fluctuation in the center wavelength of the control groups, less variance among triplicates, and more robust statistical significance compared to polydisperse sensors made with the same antibodies. Thus, chiral purification will likely enhance the function of other SWCNT sensor designs as well and therefore holds great promise for the field of optical nanosensors.

## Conclusions

In this work, we demonstrated ATPE sorting of (6,5) and (7,6) chiralities using amine-functionalized ssDNA, which is the first instance of chemically modified DNA enabling chirality purification. While the addition of the amine group altered the sorting behavior of the SWCNT within the ATPE system, our results suggest that aminated or otherwise functionalized DNA could be used to further customize sorting systems and subsequent applications of SWCNT obtained. We then performed antibody conjugation to two distinct SWCNT-DNA-NH_2_ constructs obtained from ATPE sorting, creating molecularly specific chirality-purified nanosensors. This unique sensor design combines the enhanced optical properties achieved by ATPE sorting, the high specificity enabled by antibodies, and, importantly, the opportunity for spectral multiplexing by functionalizing different (*n,m*) species with different analyte-specific recognition elements.

We are confident that the plug-and-play nature of the chirality-purified SWCNT-DNA-NH_2_ can serve as base constructs for virtually any antibody-based sensor. Future work will likely see this versatile approach utilized for other disease biomarkers, provided there is a suitable commercially available antibody to use for conjugation. The virtually unlimited library of DNA sequences and multitude of chemical modifications for oligonucleotides suggest the possibility for expansion of these methods. In addition, the application of machine learning to identify DNA sequences for chirality sorting may substantially improve the efficiency of the ATPE process^33,45^. Future studies may see the addition of aminated DNA sequences to machine learning training sets to identify more efficient sequences for sorting of SWCNT-DNA-NH_2,_ potentially coupled with molecular dynamics simulations to understand the functional group’s impact on SWCNT partitioning. Although (6,5) and (7,6) SWCNT were used in this work, this methodology could easily be expanded to additional chiralities, provided the appropriate DNA sequences are used to target different (*n,m*) species. Moreover, only two chiralities were used in this study to demonstrate SWCNT multiplexing ability, however it is certainly possible to achieve further clinical utility with additional chiralities. Hyperspectral microscopy studies have resolved 17 distinct (*n,m*) species with single nanotube spatial resolution^72^. Applying this powerful technique could enable detection of several more biomarkers, provided the sorting of the various chiralities is achieved. As the sensor platform moves forward into more challenging biologically complex environments, further testing would be required to ensure the specificity of the sensors. Ultimately, we anticipate that this work will serve as a potential launchpad for translation of implantable or bedside multiplexed diagnostic tools.

## Methods

### Preparation of SWCNT-DNA constructs

SWCNT-DNA was prepared as previously described^12^. The (6,5) sorting sequence (ss65, TTA-TAT-TAT-ATT) and (7,6) sorting sequence (ss76, (ATTT)_4_) were identified as chirality-recognizing sequences in previous ATPE studies^28,31^. Amine-functionalized versions of each sequence were also used. (TAT)_6_-NH_2_ was used as an intermediate linker for antibody conjugation on unsorted SWCNT as previously published^14–16^ (Table 1). 1 mg DNA (Integrated DNA Technologies; Coralville, IA) as added to 0.5 mg SWCNT powder (SG65i [Sigma-Aldrich; Burlington, MA], SG76 [Sigma-Aldrich; Burlington, MA], or HiPCO [Nanointegris; Boisbriand, Quebec]) in 0.5 mL deionized water with 0.1 M NaCl. Samples were sonicated on ice at 40% amplitude for 1 h by a VCX 750 ultrasonicator with a 2 mm stepped microtip probe (Sonics & Materials, Inc.; Newtown, CT). Sonicated suspensions were ultracentrifuged at 58,000 × *g* for 1 h using an Optima Max-XP Ultracentrifuge (Beckman Coulter; Brea, CA). The top 75% of the suspension was collected for further use. Samples were stored at 4 °C for up to 14 days. Within 24 h of use, samples were filtered to remove free DNA. An aliquot of SWCNT-DNA was loaded into a 100 kDa MWCO centrifugal filter (Sigma-Aldrich; Burlington, MA) and centrifuged for 15 min at 14,000 × *g*. Filtrate was discarded, and the contents of the filter were resuspended in deionized water, then centrifugally filtered again. The retained content was collected and resuspended in deionized water for further analysis. All subsequent experiments were performed in aqueous solution.

**Table 1:** ssDNA Sequences Used for SWCNT Dispersion

Name	Sequence
ss65: (6,5) sorting sequence^31^	TTA-TAT-TAT-ATT
ss65-NH_2_: aminated (6,5) sorting sequence	TTA-TAT-TAT-ATT-NH_2_
ss76: (7,6) sorting sequence^74^	(ATTT)_4_
ss76-NH2: aminated (7,6) sorting sequence	(ATTT)_4_-NH_2_
(TAT)_6_-NH_2_: intermediate linker sequence^14–16^	(TAT)_6_-NH_2_

### Synthesis of ATPE systems and mimics

ATPE systems were prepared as described by others^31^. 400 μL ATPE systems were made with 57 μL of 60% (m/m) 1.5 kDa polyethylene glycol (PEG) (Sigma-Aldrich; Burlington, MA), 202 μL of 20% (m/m) 250 kDa Dextran (DEX) (Alfa Aesar; Haverhill, MA), 21 μL of deionized water, and 120 μL of ~ 500 mg/L SWCNT-DNA or SWCNT-DNA-NH_2_. As ATPE is a scalable technique, 3200 μL systems were prepared using the same ratio of reagents with volumes multiplied 8x^18^.

Similarly, the ATPE mimic used the same amount of PEG and DEX but instead replaced the SWCNT dispersion with the identical volume of water. For our experiments, we chose to scale the mimic 30x from the ATPE system in order to have sufficient polymer volume for our separations. The mimic was vortexed for 1 min and centrifuged at room temperature for 5 min at 3200 × *g* to allow for phase (PEG/DEX) separation.

### ATPE separation of SWCNT

2 μL of 1% polyvinylpyrrolidone (PVP, 10 kDa, Sigma-Aldrich; Burlington, MA) was initially added to the 400 μL ATPE system. We vortexed the system for 1 min and centrifuged at 3200 × *g* for 5 min or until a clear phase separation was visible. After centrifugation, the top fraction of the ATPE system was extracted via micropipette and saved for optical characterization. The extracted top phase was replaced with an equal volume of the top phase of the mimic solution to keep total volume and polymer concentrations consistent throughout the chirality sorting process. 2 μL 1.25% PVP was added along with the mimic. The ATPE system was then vortexed and centrifuged as before. The top phase was again extracted and saved for optical characterization. This process was repeated, with the addition of 2 μL 1.25% PVP each iteration, until the purified target chirality was present, whether it be in the top or bottom phase. Only when sorting with ss76 or ss76-NH_2_ was 2 μL 2% PVP used starting with the 4th extraction and continuing until the end.

### Optical characterization of SWCNT

At each step of the ATPE process, SWCNT fractions were subjected to absorbance measurements (V-730 UV–vis Spectrophotometer, Jasco; Easton, MD) from 300–1100 nm using a with a 400 nm min^−1^ scan rate and 0.2 nm steps. The concentration of SWCNT was determined using the equation: C = A_630_/0.036 l mg^−1^ cm^−1^ * DF, where DF = dilution factor^73^. We also performed NIR fluorescence spectroscopy on each SWCNT fraction using a NS MiniTracer (Applied NanoFluorescence; Houston, TX) using a 50 mW 638 nm laser in the range of 900–1600 nm. After optical characterization was complete, samples were kept in the 4 °C fridge until use.

### Engineering molecularly-specific sensors

We then conjugated cytokine-specific antibodies to the chirally-sorted SWCNT-DNA-NH_2_ samples via carbodiimide conjugation chemistry^14–16^. Separately, as a non-separated comparison, antibody conjugation was also performed on HiPCO (bulk chirality) SWCNT wrapped with (TAT)_6_-NH_2_. IL-6 (RRID: AB_398568, BD Biosciences; Franklin Lakes, NJ) and IL-12 (RRID: AB_2929120, PeproTech; Cranbury, NJ) antibody conjugations were performed separately. The carboxylic acids of the antibody were first activated with 1-ethyl-3-(3-dimethylainopropyl) carbodiimide (Fisher Scientific; Hampton, NH) and N-hydroxysuccinimide (Fisher Scientific; Hampton, NH) in a 10X and 25X molar excess, respectively, for 15 min. This reaction was quenched with 1 μL of 2-mercaptoethanol (Fisher Scientific; Hampton, NH). The activated antibody was added in an equimolar ratio to the DNA, assuming a 1:1 ratio of ssDNA to SWCNT. Following 2 h of incubation on ice, the conjugate was dialyzed against water with a 1000 kDa MWCO SpectraPor Float-A-Lyzer (Repligen; Waltham, MA) at 4 °C for 48 hours with two buffer changes to remove unconjugated antibody, reaction reagents, and leftover ATPE polymers from the solution. The resulting sensors—made from IL-6 antibody conjugated to (7,6)-sorted SWCNT (IL-6 Ab-(7,6)) and IL-12 antibody conjugated to (6,5)-sorted SWCNT (IL-12 Ab-(6,5))—were collected from dialysis and stored at 4 °C. After sample collection, we performed light scattering measurements to confirm successful antibody conjugation. Dynamic light scattering was used to measure the particle size before and after conjugation, while electrophoretic light scattering was performed to compare the relative zeta potential of the particles before and after conjugation (Nano-ZS90, Malvern; Worcestershire, U.K.).

Further characterization was performed via atomic force microscopy (AFM). (7,6)-purified SWCNT-DNA-NH_2_ with or without IL-6 antibody conjugation were subjected to three additional Amicon filtrations to concentrate the sample and remove excess ssDNA. Samples were deposited onto freshly cleaved mica substrates at a concentration of 0.5 mg/L and allowed to dry completely before imaging. AFM was performed using a Bruker MultiMode 8 system (Billerica, MA). Imaging was conducted in air with a scan size of 1 µm × 1 µm at a scan rate of ~2 Hz and a resolution of 512 × 512 pixels. AFM data were processed using the NanoScope Analysis 1.8 software.

### Sensor characterization

All sensor evaluations were performed in 1X PBS. Prior to use, sensors were passivated by incubation with 50x mass ratio poly-L-lysine (PLK, Advanced BioMatrix; Carlsbad, CA) for 30 min at 4 °C, as previously-described to direct analyte binding to the antibody rather than the exposed SWCNT surface^16^. We incubated 0.5 mg/L SWCNT sensors with 5 μg/mL of the target cytokine protein (Human IL-6 Recombinant Protein, Gibco; Waltham, MA **or** Recombinant Human IL-12 (linked heterodimer), R&D Systems; Minneapolis, MN) in 1X PBS to confirm functionality. NIR fluorescence spectra were obtained with ClaIR plate reader (Photon Etc; Montreal, Quebec) in 15-minute intervals for 3 h following protein addition.

To evaluate multiplexing ability, an equal mass of IL-6 Ab-(7,6) and IL-12 Ab-(6,5) sensors were combined into one sample. The combined sensors were tested against IL-12 individually, IL-6 individually, as well as both cytokines combined. The specificity of the sensors was further challenged by testing them against non-target cytokines IL-1β and TNF-α. Sensor dynamic range was investigated using a broad range of analyte concentrations (from 15 pg/mL to 10 μg/mL). All sensor samples were prepared in triplicate unless otherwise specified.

### Data analysis

NIR fluorescence corresponding to individual nanotube chirality emission peaks was fit with a custom MATLAB code to a Voigt model to determine their center wavelength and maximum intensity values (MATLAB code is available upon request). Changes in the SWCNT center wavelength and maximum intensity values were reported relative to their baseline emission prior to antigen addition. Means for triplicate samples plus the standard deviation were obtained. Error bars on graphs represent standard deviation from the means. Statistical significance was determined with a two-sample *t* test. * *p* < 0.05, ** *p* < 0.01, *** *p* < 0.001.

## Supplementary information

Supplementary Material

## Data Availability

Datasets generated and analyzed during this study include near infrared fluorescence spectral data and atomic force microscopy images. All data supporting the findings of this study are included in this published article and its supplementary information files. Additional raw and processed datasets are available from the corresponding author upon request.

## Code availability

Near infrared fluorescence spectra corresponding to individual nanotube chirality emission peaks were fit with custom MATLAB code to a Voigt model to determine their center wavelength and maximum intensity values. MATLAB code is available from the corresponding author upon request.
