# Supplementary material for: Antibody-conjugated species-sorted single-walled carbon nanotubes for multiplexed cytokine sensing
Source: Commun Mater. 2026 Apr 27;7(1):167. doi: 10.1038/s43246-026-01169-y (PMC13221203; doi:10.1038/s43246-026-01169-y)
Supplement: Supplementary file 1 — Supplementary Material [file 43246_2026_1169_MOESM1_ESM.pdf]

Supplementary Information for:

Antibody-conjugated species-sorted single-walled carbon nanotubes for multiplexed cytokine sensing

Amelia K. Ryan[1], Sadiyah Parveen[1], Zachary Cohen[1], Atara Israel[1,2], and Ryan M. Williams*[1,2]

[1]The City College of New York, Department of Biomedical Engineering, New York, NY, USA

[2]Stony Brook University, Department of Medicine, Division of Nephrology & Hypertension, Stony Brook, NY, USA

*ryan.williams@stonybrookmedicine.edu

Supplementary Methods

Supplementary Figures S1-S10

Supplementary Tables S1-S3

Supplementary Methods:

ATPE Sorting of SWCNT-DOC: To prepare DOC-SWCNT systems, 1.5 mg SWCNT powder (SG65i [Sigma-Aldrich; Burlington, MA], SG76 [Sigma-Aldrich; Burlington, MA], or HiPCO [Nanointegris; Boisbriand, Quebec]) was dispersed in 1.5 mL of aqueous 1.0% (m/v) sodium deoxycholate (DOC [Sigma-Aldrich; Burlington, MA]). SWCNT-DOC were sonicated as previously described and ultracentrifuged at 58,000 X g for one hour. After centrifugation, the supernatant dispersion was collected, as previously described, for further analysis.

The SWCNT-DOC ATPE systems were synthesized by mixing 2 mL of DI water with 0.8 ml of 40% (m/m) 6 kDa PEG (Alfa Aesar; Ward Hill, MA), 0.8 ml of 20% (m/m) 70 kDa DEX (TCI; Portland, OR), 0.2 ml of 10% (m/v) sodium dodecyl sulfate (Fisher Scientific; Waltham, MA), and lastly 0.2 ml of SWCNT-DOC. Using the same polymer solutions, the mimic was synthesized by mixing 20 ml of DI water with 8 ml of 40% PEG, 8 ml of 20% DEX, 2 ml of 10% SDS, and 2 ml of 1% (m/v) DOC. The mimic was vortexed for 1 minute and centrifuged at room temperature for 5 mins at 3,200 Xg to allow for phase (PEG/DEX) separation.

A certain volume of 0.5M hydrochloric acid (HCl, Fisher Scientific; Waltham, MA) was added to the ATPE system (**Table S1**). The system was vortexed for 1-2 mins and centrifuged at 3,200 Xg for 5 mins. After centrifugation, the first top phase (T1) was removed and replaced with an equal volume of the top phase of the mimic. An additional volume of 0.5 M HCl was added, followed by the same mixing and centrifugation process as previously mentioned. The second top phase (T2) was removed and collected, and this process was repeated until the target chirality moved to the top phase (Tx). Keeping Tx, the bottom phase was removed and replaced with an equal amount of the bottom phase of the mimic. After, a certain volume of sodium hypochlorite (NaClO, Sigma-Aldrich; Burlington, MA) and a predetermined volume of 0.5 M HCl were added to the system, followed by mixing and centrifugation, the purified target chirality moved to the bottom phase.

Table S1: Volumes of HCl and NaClO added at each step of ATPE sorting of SWCNT-DOC.

**Target chirality**	**Volume of 0.5 HCl (μL) added at each step**			
	**T1**	**T2**	**T3**	**T4**
(6,5)	5.5	6.5	7.5 $^{(1)}$	-------
(7,6)	2.5	3.5	4.2	4.8 $^{(2)}$
(1) 20 μL NaClO with 5.7% available chloride and (2) 10 μL of NaClO with 10-15% available chloride added				

Surfactant Exchange: After ATPE sorting was finished, a surfactant exchange was performed to switch DOC for DNA-NH_2. 50 μL SWCNT in 1% DOC solution was combined with 10 μL of either ss65-NH_2 or ss76-NH_2. The solution was vortexed while 100 μL of methanol was slowly added with a syringe. According to previously published protocols, the solution should appear homogenous after the addition of methanol. However, our solutions consistently appeared heterogenous with SWCNT aggregates and showed no observable fluorescence spectra.

10 µL 5M NaCl (in deionized water) was added to precipitate SWCNT. However, the SWCNT could not be homogenously resuspended. This method was therefore not used for further experiments.

Challenging Unconjugated SWCNT with Cytokines: To evaluate the role of the antibody in sensor response and evaluate the effects of nonspecific protein adsorption to the SWCNT surface, we deployed chirality-sorted, PLK-passivated SWCNT-ss76-NH_2 or SWCNT-ss65-NH_2 against 5 µg/mL (the same concentration as in Figures 3-5) IL-6 or IL-12, respectively. NIR fluorescence measurements and analysis was performed as described in the main text.

Antibody Conjugation for Alternative Analytes:

Additional antibody conjugations were performed using primary amine-functionalized ssDNA-sorted SWCNT. Using the same conjugation methods described in the main text, IL-8 antibody (Clone 3IL8-H10, Invitrogen; Waltham, MA) was conjugated to (6,5) sorted tubes and IL-1β antibody (Accession #PA5119221, Invitrogen; Waltham, MA) was conjugated to (7,6) sorted tubes. The sensors were then combined and tested against IL-8 (Accession #P10145, Gibco; Waltham, MA) and IL-1β (Accession #P01584, Gibco; Waltham, MA) recombinant proteins using the same methods described in the main text.

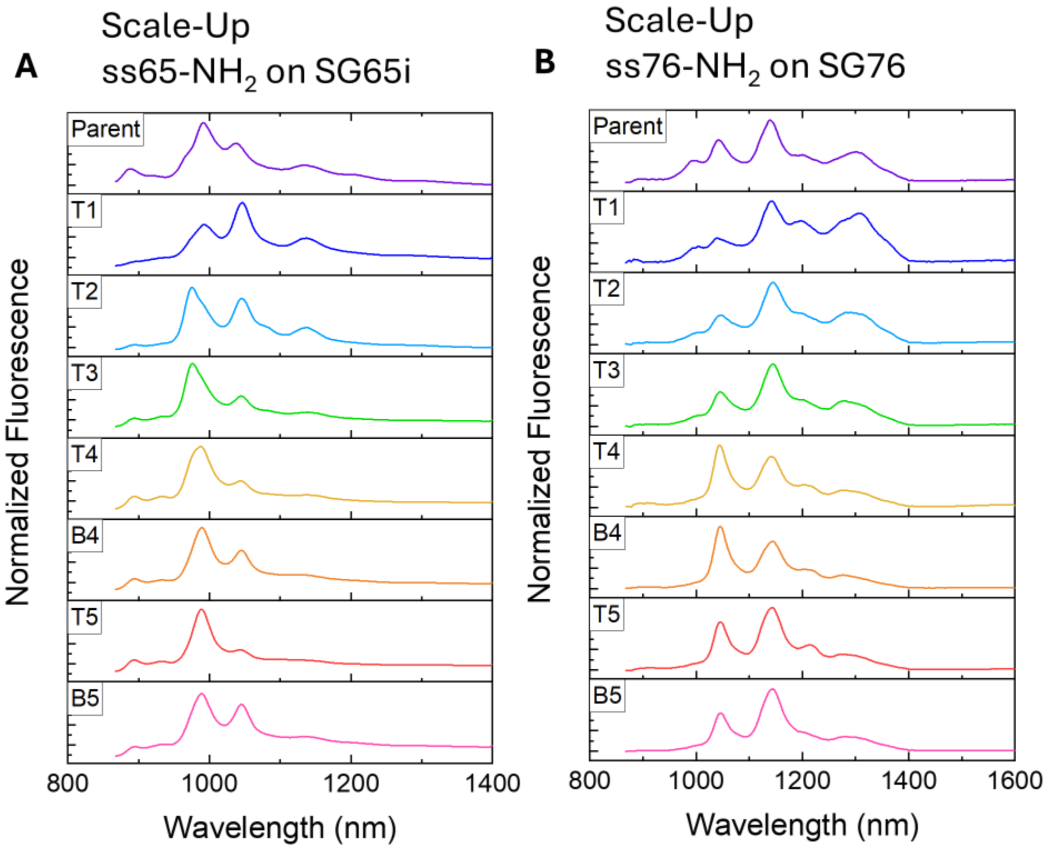

Figure S1: Scale-up of ATPE sorting of SWCNT-DNA-NH_2. A) Fluorescence spectra from each phase of ATPE sorting of SG65i using ss65-NH_2. **B)** Fluorescence spectra from each phase of ATPE sorting of SG76 using ss76-NH_2 (excitation wavelength = 638 nm).

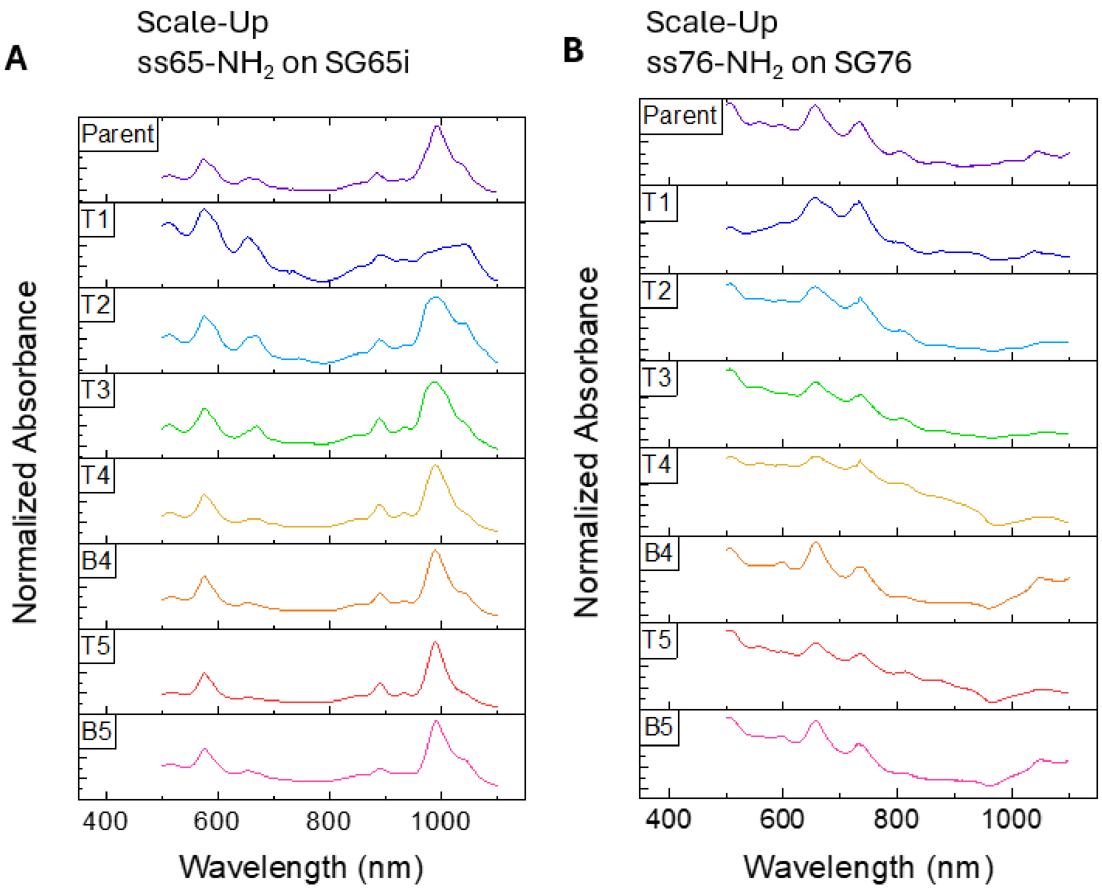

Figure S2: Scale-up of ATPE sorting of SWCNT-DNA-NH_2. A) Absorbance spectra from each phase of ATPE sorting of SG65i using ss65-NH_2. **B)** Absorbance spectra from each phase of ATPE sorting of SG76 using ss76-NH_2.

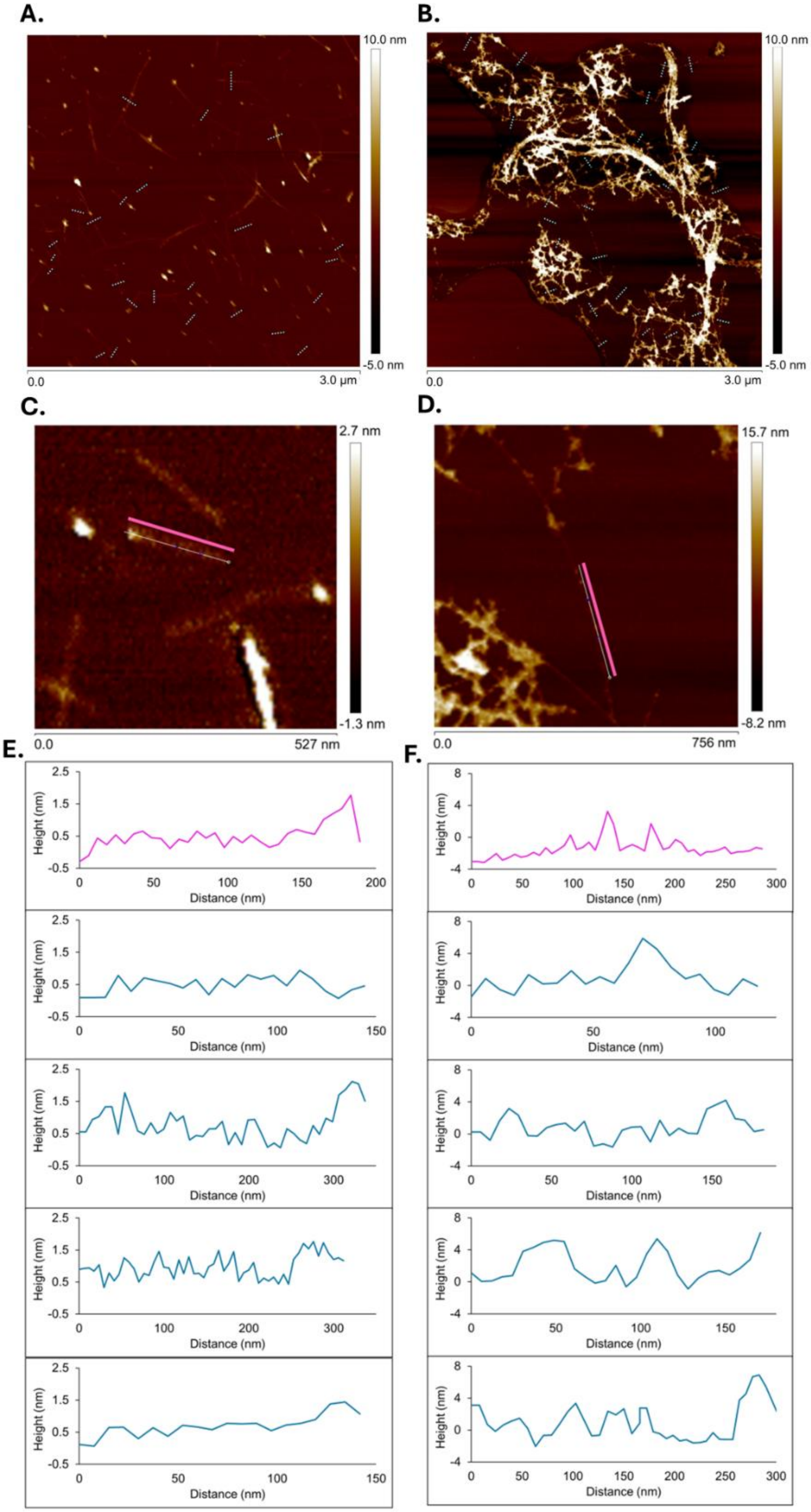

A.
10.0 nm
-5.0 nm
0.0
3.0 µm
B.
10.0 nm
-5.0 nm
0.0
3.0 µm
C.
2.7 nm
-1.3 nm
0.0
527 nm
D.
15.7 nm
-8.2 nm
0.0
756 nm
E.
F.
Height (nm)
Distance (nm)

Figure S3: Selection of nanotubes for AFM height analysis. A) AFM image of SWCNT-ss76-NH_2 with all perpendicular cross-sections used for height analysis indicated (n = 25). **B)** AFM image of IL-6 Ab-(7,6) with cross-sections used for height analysis indicated (n = 25). **C)** Longitudinal height profiles of SWCNT-ss76-NH_2. **D)** Longitudinal height profiles of IL-6 Ab-(7,6).

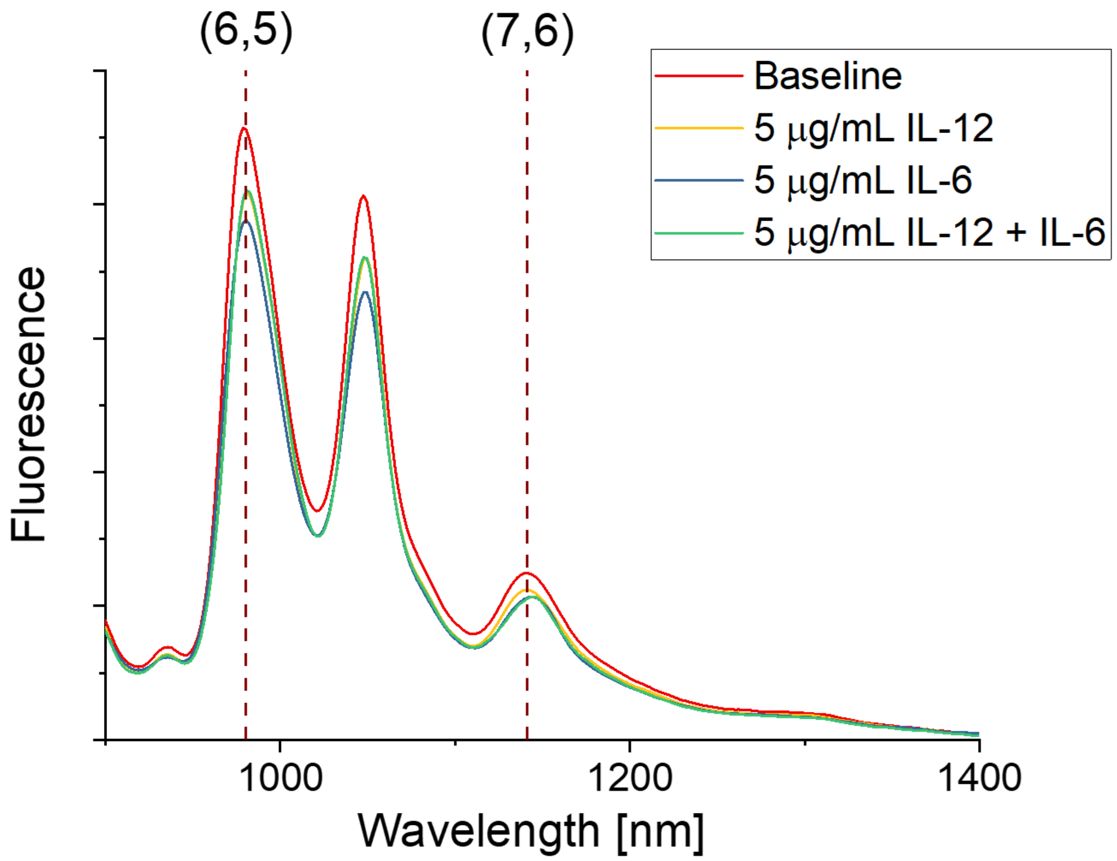

Figure S4: NIR fluorescence spectra of individual and multiplexed cytokine detection (acquired with 655 nm laser).

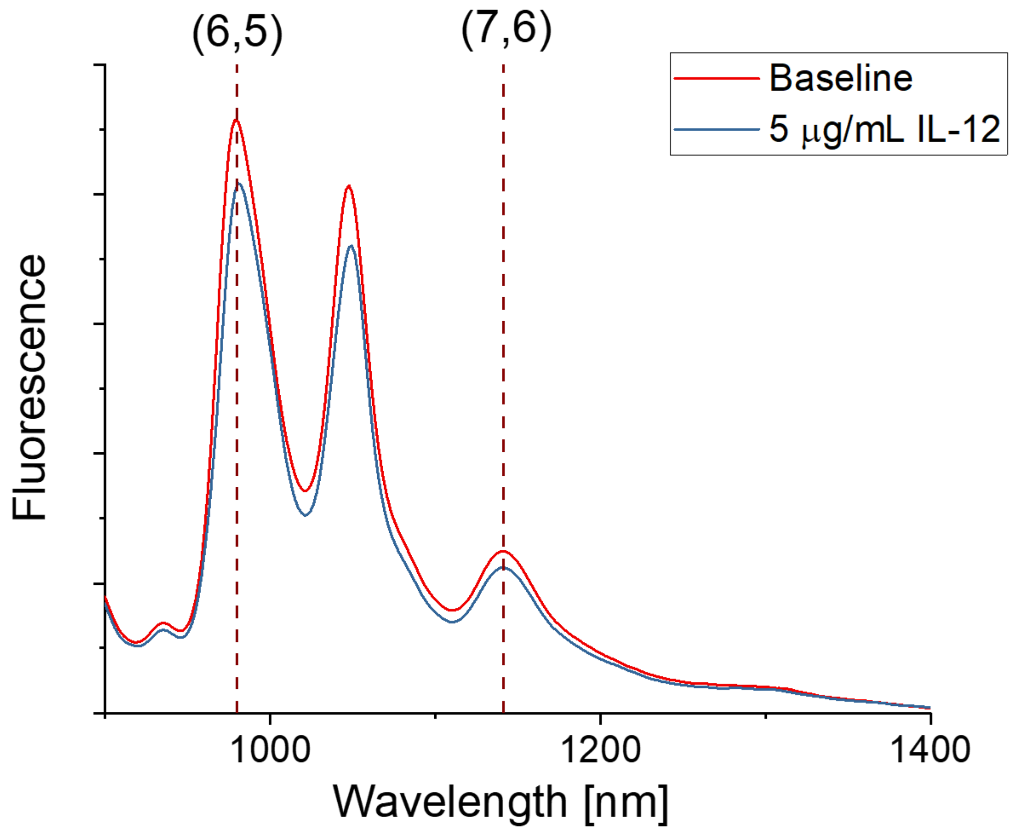

Figure S5: NIR fluorescence spectra of IL-12 detection (acquired with 655 nm laser).

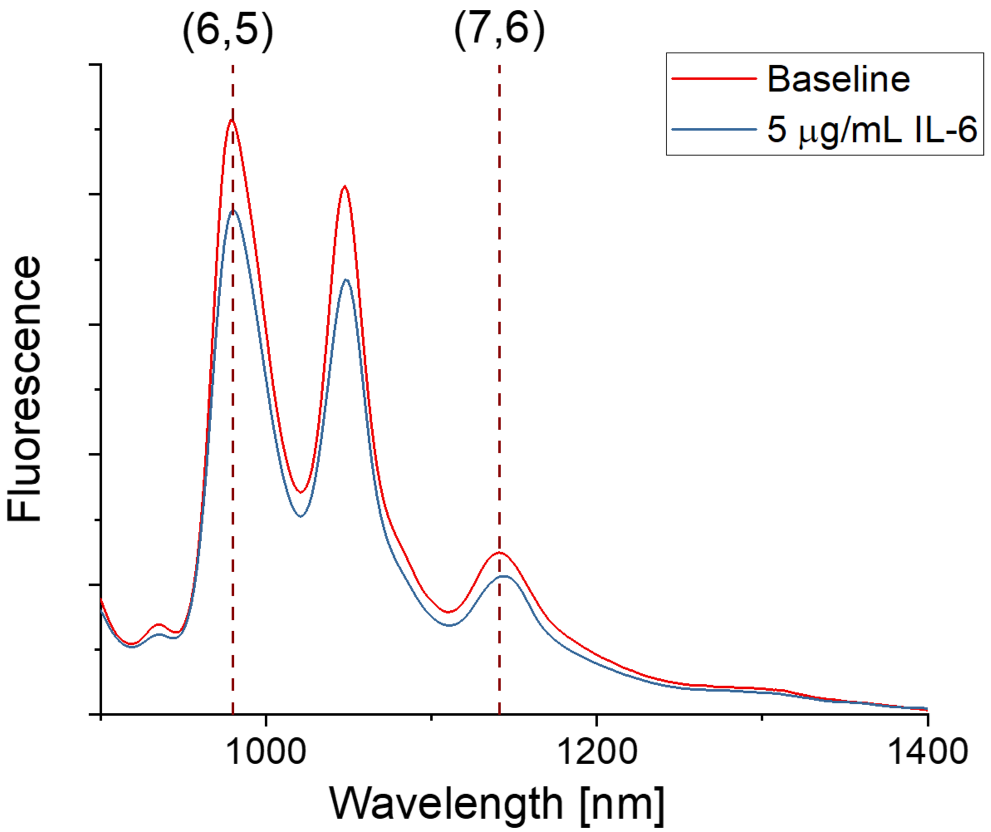

Figure S6: NIR fluorescence spectra of IL-6 detection (acquired with 655 nm laser).

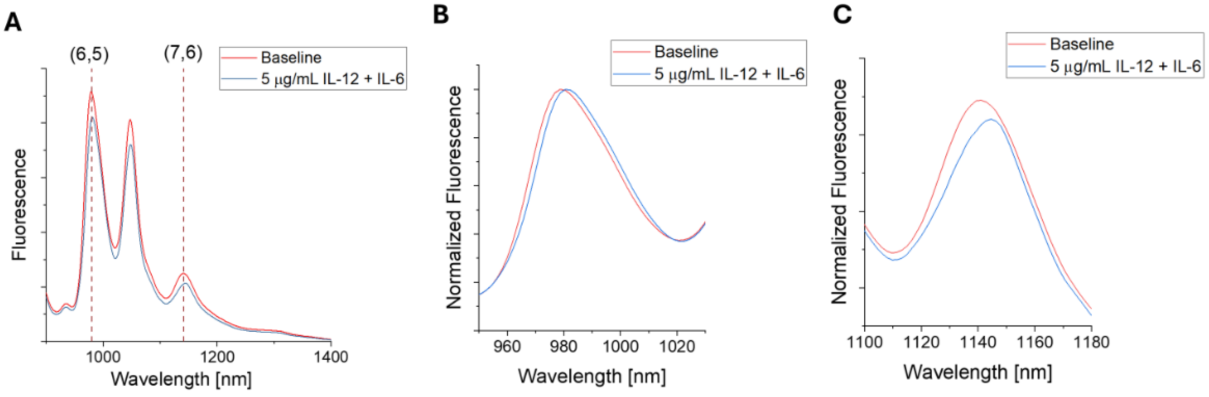

Figure S7: NIR fluorescence spectra of simultaneous IL-12 and IL-6 detection. A) Full NIR spectrum (acquired with 655 nm laser). B) (6,5) peak spectral shift. C) (7,6) peak spectral shift.

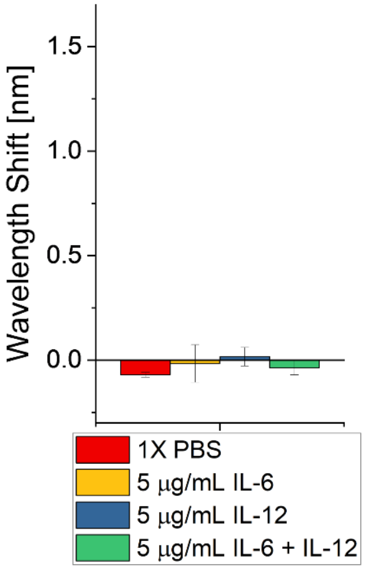

Figure S8: Wavelength shift of (7,5) peak in combined sensor samples response to addition of cytokines (IL-6: $p = 0.183$. IL-12: $p = 0.742$. IL-6 + IL-12: 0.180).

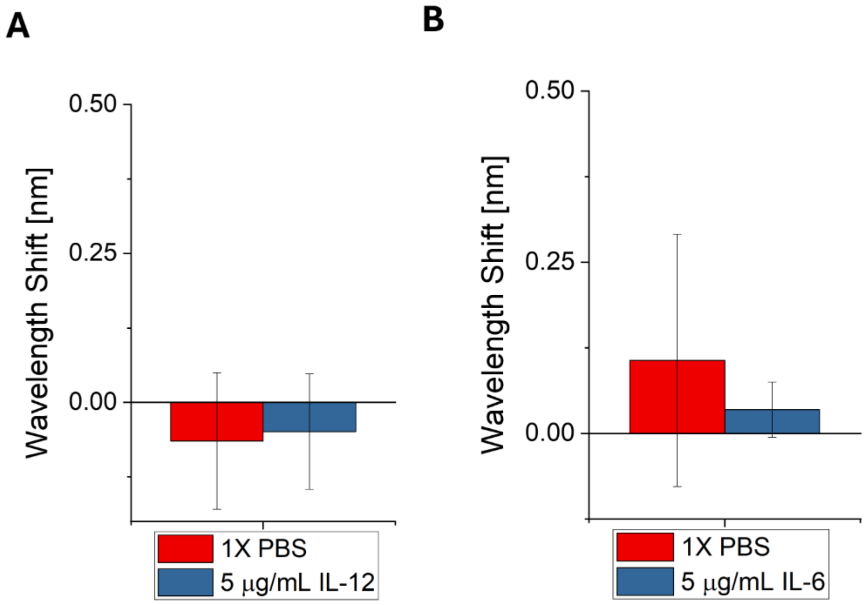

Figure S9: Response of unconjugated SWCNT-DNA-NH_2 to cytokines. A) Shift of (6,5) peak of SWCNT-ss65-NH_2 in response to IL-12 addition ($p = 0.867$). B) Shift of (7,6) peak of SWCNT-ss76-NH_2 in response to IL-6 addition ($p = 0.543$).

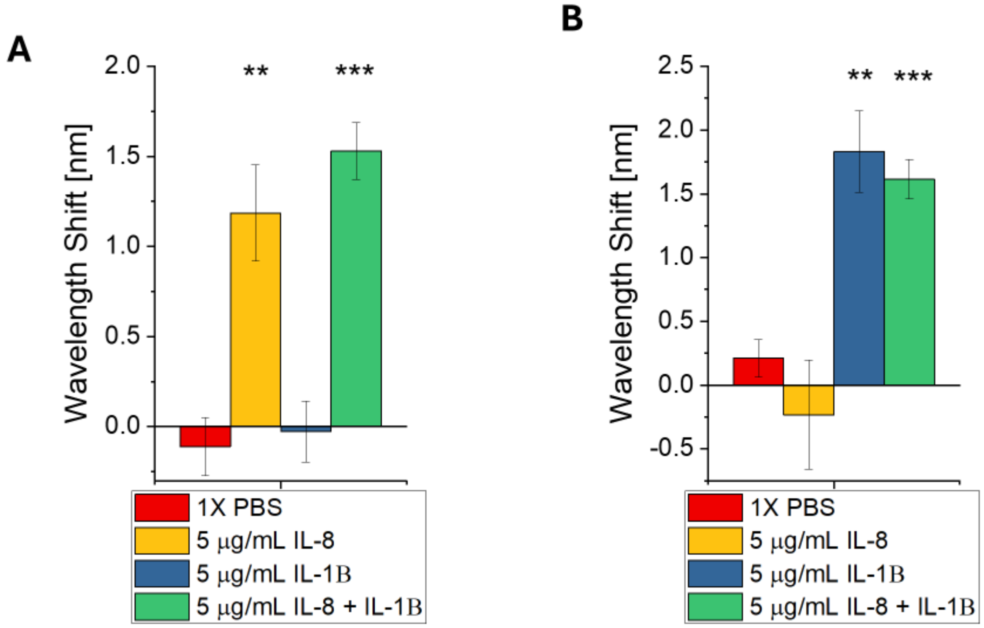

Figure S10: Multiplexed sensing of IL-8 and IL-1β. A) Wavelength shift (6,5) peak of combined sensor samples in response to cytokine addition (IL-8: $p = 0.00198$. IL-1β: $p = 0.569$. IL-8 + IL-1β: $p = 0.000229$). B) Wavelength shift (7,6) peak of combined sensor samples in response to cytokine addition (IL-8: $p = 0.166$. IL-1β: $p = 0.00132$. IL-8 + IL-1β: $p = 0.000339$).

Table S2: (6,5) concentration-response curve p values.

Cytokine Concentration (pg/mL)	IL-6 (denoted by *)	IL-12 (denoted by †)	IL6 + IL-12 (denoted by ‡)
1.05E7	0.4749	1.16249E-4	2.82828E-5
5000000	0.0155	2.857E-4	0.00274
1500000	0.5484	5.674E-5	8.933E-5
500000	0.5861	9.838E-4	7.901E-5
50000	0.5887	2.057E-4	1.916E-4
15000	0.2107	8.402E-4	4.592E-4
5000	0.0949	2.965E-4	9.284E-4
1500	0.6815	7.2778E-4	5.319E-4
150	0.8251	7.308E-4	0.00176
15	0.693	0.00744	0.00358

Table S3: (7,6) concentration-response curve p values.

Cytokine Concentration (pg/mL)	IL-6 (denoted by *)	IL-12 (denoted by †)	IL6 + IL-12 (denoted by ‡)
1.05E7	1.75642E-6	0.00872	4.44266E-5
5000000	3.607E-5	0.02102	9.665E-6
1500000	2.0618E-5	0.63889	3.967E-5
500000	0.00313	0.00331	1.945E-4
50000	1.609E-5	0.02241	1.692E-5
15000	3.375E-5	0.80408	4.220E-5
5000	2.468E-4	0.35082	6.509E-6
1500	2.792E-4	0.77577	2.671E-4
150	5.298E-4	0.14737	9.998E-4
15	9.858E-4	0.23596	7.024E-4
